# "Sanitas" Oil and Inunction

**Published:** 1893-08-05

**Authors:** C. T. Kingsett

**Affiliations:** Elmstead Knoll, Chislehurst, Kent


### EDITOR'S LETTER-BOX.

[Our correspondents are reminded that prolixity is a great bar to publication, and that brevity of style and conciseness of statement greatly facilitate early insertion.]

### "SANITAS" OIL AND INUNCTION.

SIR,—In his last letter to you Dr. Curgenven evades all the real issues and takes no notice of my challenge to him, from which I can only conclude he has not the courage of his reported opinion. I repeat that "Sanitas" oil is a more powerful antiseptic and germicide than any variety of eucalyptus oil, and one of its active principles is the substance which Dr. Curgenven erroneously writes of as "resin," and which is left on paper after the more volatile part of the oil has evaporated. The mere fact that "Sanitas" oil leaves such a residue, while eucalyptus oil is entirely volatile, only proves that the latter substance is devoid of this very important active ingredient, the presence of which, instead of making "Sanitas" oil unfit for inunction, the better qualifies it, on the other hand, for such employment.

I should add that "Sanitas" oil is a pure drug, and does not contain any added substance as Dr. Curgenven's letter seems to imply. Nothing that Dr. Curgenven has yet published has proved "Sanitas" oil to be unfit for such use in any way. He has only printed unfounded hypothetical statements with reference to "Sanitas" oil, with the chemistry of which he is evidently entirely unacquainted, although I have published a perfect "array of facts" such as he asks for on the subject. He will find this information summarised in my work on "Nature's Hygiene," third edition (Baillière and Co.).—Yours truly.

C. T. KINGSETT, F.I.C., F.C.S.
(per C. W. Harris, Sec.)

Elmstead Knoll, Chislehurst, Kent,

July 31st, 1893.

[*₊* It is but fair to state that Dr. Curgenven enclosed specimen papers of the action of "Sanitas" and other oils, which we have sent to Dr. Kingsett.—ED. *T. H.*]

